# A machine learning approach towards the prediction of protein–ligand binding affinity based on fundamental molecular properties†

**DOI:** 10.1039/c8ra00003d

**Published:** 2018-03-28

**Authors:** Indra Kundu, Goutam Paul, Raja Banerjee

**Affiliations:** Department of Bioinformatics, Maulana Abul Kalam Azad University of Technology (formerly known as West Bengal University of Technology) Kolkata India indraknd@gmail.com; Indian Statistical Institute Kolkata India goutam.k.paul@gmail.com; Maulana Abul Kalam Azad University of Technology (formerly known as West Bengal University of Technology) Kolkata India banraja10@gmail.com

## Abstract

There is an exigency of transformation of the enormous amount of biological data available in various forms into some significant knowledge. We have tried to implement Machine Learning (ML) algorithm models on the protein–ligand binding affinity data already available to predict the binding affinity of the unknown. ML methods are appreciably faster and cheaper as compared to traditional experimental methods or computational scoring approaches. The prerequisites of this prediction are sufficient and unbiased features of training data and a prediction model which can fit the data well. In our study, we have applied Random forest and Gaussian process regression algorithms from the Weka package on protein–ligand binding affinity, which encompasses protein and ligand binding information from PdbBind database. The models are trained on the basis of selective fundamental information of both proteins and ligand, which can be effortlessly fetched from online databases or can be calculated with the availability of structure. The assessment of the models was made on the basis of correlation coefficient (*R*^2^) and root mean square error (RMSE). The Random forest model gave *R*^2^ and RMSE of 0.76 and 1.31 respectively. We have also used our features and prediction models on the dataset used by others and found that our model with our features outperformed the existing ones.

## Abstract 2

There is an exigency of transformation of the enormous amount of biological data available in various forms into some significant knowledge.

## Graphical abstract

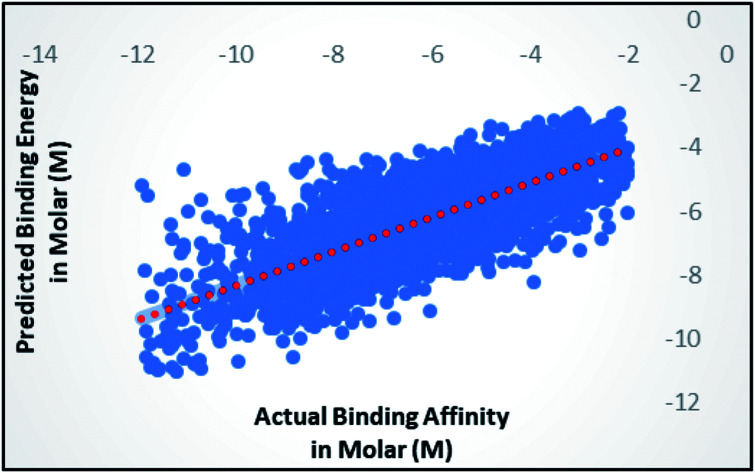

## Introduction

The cardinal goal of drug discovery is to design and deliver selective compounds against individual biological targets. In general, it takes about 15 years and up to 800 million dollars to convert a promising new compound into a drug.^1^ The approaches and methodologies used in drug design have been changed over time. In an early stage of the drug discovery process, the focus is on reducing the number of drug candidates and this problem has been deciphered using computational approaches.^2^ A drug is a small molecule which activates or inhibits the function of protein, as proteins are one of the popular targets for the drug designing process.^3^ The interaction between a protein and ligand is specific. These specific molecular interactions between proteins and its ligand plays crucial role to a broad spectrum of biological functions.^4–6^ Predicting interactions between ligand and proteins is an indispensable element in the drug discovery process.^3,7^

In order to perform a rapid search for molecules that may bind to targets of biological interest computational techniques such as structure based drug designing (SBDD) is carried out, which includes structure based virtual screening (SBVS) or molecular docking followed by Molecular Dynamics.^3,8,9^ Molecular docking is one of the most frequently used methods because of its ability to predict the conformation and affinity of ligand binding to the target site, with a substantial degree of accuracy.^10,11^ Docking methods effectively search high-dimensional spaces for plausible interaction and use a scoring function that correctly ranks the candidate.^12^ Although the results of docking are specific and reliable; however, screening of umpteen molecules maneuvering every step of docking can be wearisome. As docking is a time consuming process, it could be worth it if some faster methods can be employed to predict whether a molecule can bind the biologically active target molecule to initiate the biological function. Towards the end, Machine Learning (ML)^13^ techniques can be an alternative choice.

Machine learning algorithms build a model from training inputs in order to make data-driven predictions or decisions, expressed as outputs.^13,14^ These methods will statistically analyze the correlation between chemical structures and interaction status of known protein and ligand pairs to derive statistical models for predicting the status of other unknown compounds.^15^ It does not demand the explicit program for the learning procedure of the machine. Supervised prediction can be based on either classification or regression.^16^ Classification is used when the discrete value is to be predicted, whereas regression is used where the values are diverse and cannot be predicted exactly hence, accuracy is measured on the basis of closeness of predicted value to true value. Prediction of binding energy value requires regression algorithm and predicting the feasibility of interaction can be fulfilled by classification. Other than this, statistical learning method has recently been used for classification of G-protein coupled receptors and DNA-binding proteins. It has also been employed in a number of other protein structure, interaction prediction studies including fold recognition,^17^ protein–protein interaction prediction,^18,19^ solvent accessibility^20^ and structure prediction.^21^

However, studies combining the spheres of protein–ligand interactions and machine learning conducted till date were mostly focused on a particular protein or a particular class of proteins. Laurent Jacob *et al.* carried out a study involving targets with no or few known ligands and succeeded in predicting enzymes and GPCR with an accuracy of 86.2% and 77.6% respectively.^22^ Masayuki Yarimizu *et al.* accomplished a study using Support Vector Machine (SVM) on tyrosine receptor and predicted whether a molecule is a ligand of the tyrosine receptor or not with a very high accuracy, AUC was 0.996.^23^ The study is focused on tyrosine kinases. Laurent Jacob *et al.*^22^ and Masayuki Yarimizu *et al.*^23^ used ML for classification, where there is binary class *i.e.* yes or no. For prediction of discrete value ML regression is employed.^13,14,16^ Xue *et al.*^24^ utilised regression for prediction of binding energy in terms of log *K* using SVM models, their study was focused on drugs against a single protein, human serum albumin.

In order to utilize the application of ML in much broader aspect beyond a particular protein or particular class of proteins, in this paper, we have addressed this issue over a heterogeneous class of proteins with variety of ligands and trained the machine so as to predict the preferable interaction through calculation of binding energy. To the best of our knowledge, such an effort would be reported for the first time. We have used Weka 3.6.8,^16,25,26^ which is a popular data mining tool that provides various machine learning algorithms.

Receptors which were diverse in their molecular function, biological process, and cellular component were considered. Deng *et al.*^27^ used a diverse dataset of 105 protein–ligand complexes, Kramer and Gedeck^28^ used pdbbind version 2009, whereas Wang *et al.*^29^ did a wide range study on pdbbind benchmark version 2007 and 2012. We have used pdbbind dataset version 2015 for our study in which we obtained correlation coefficient (*R*^2^) and root mean square error (RMSE) 0.76 and 1.31 respectively. We have also tested our model and features on the protein–ligand list used by Wang *et al.*,^29^ Deng *et al.*,^27^ Xue *et al.*^24^ and Kramer and Gadeck^28^ and have successfully recorded a better correlation coefficient of 0.75, 0.75, 0.86, and 0.72 than the reported 0.67, 0.64, 0.63 and 0.69 respectively.

## Materials and methods

### Dataset

All the protein–ligand binding affinity data were acquired from the PdbBind Database.^30,31^ The database (v2015) comprises of binding energy for all types of biomolecular complexes available in RCSB.^32^ Hence, it bridges structural information with the binding affinity of complexes. We have focused only on the protein–ligand complexes, excluding protein–nucleic acid and protein–protein complexes. There were 11 987 instances of protein and ligand. Binding affinity data was available in terms of *K*_i_ (inhibition constant), *K*_d_ (dissociation constant), IC_50_, EC_50_. We have taken into account the instances having activity in terms of either *K*_i_ or *K*_d_, rejecting those which have the activity in terms of assay dependent IC_50_ or EC_50_. The dataset was further refined by excluding the ligands which had incomplete structure. Proteins which bind to isomers, peptide like compounds, and more than one compound or have metal ions as cofactors were also eliminated. Our final dataset consists of 2864 instances, which comprises proteins of diverse class listed in ESI S1.† There, we have given the classification of the proteins used by us on the basis of their functions. They are of diverse types and the data is non-redundant in the sense that no two rows are exactly the same – either the protein or the ligand is different. Training and testing set are assigned randomly but we have also reported blind set validation.

Our primary goal was to develop a tool which can be used in the very initial stage of drug discovery process for predicting potential candidates. As diverse drugs act on a diverse set of enzymes, therefore we focused on training machine on heterogenous class of protein which are both functionally and structurally diverse. We have used pdbbind version 2015 for our study. Wang *et al.*^29^ did a similar study using 2012 version but they did not eliminate isomers or incomplete ligand structure, whereas we have included even the low-resolution structure. Despite the dataset being created from the same source our dataset varies a lot listed in ESI S1.† We have used their exact training and test dataset for one generic and 3 family specific dataset namely HIV protease, trypsin, carbonic anhydrase with our features. Other than this we have also compared our features on the dataset used by Deng *et al.*^27^ and Xue *et al.*^24^

Our dataset has 2864 rows and 128 columns. The rows represent the protein–ligand pair whereas the columns are their properties. Each row of the Dataset can be represented as ***X***1_1_, ***X***2_1_, …, ***Y***1, where ***x*** are the features and ***y*** is the class that will be predicted by our models. Our Dataset can be represented as follows
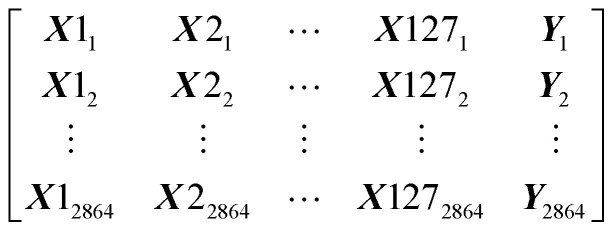


Machine learning algorithms will find the pattern which will fit *x* and create a function *f*(*x*) that can predict *y* for a new *x*.

### Features extraction

Training a machine is highly dependent on features. Feature selection must be done with utmost care. Drug binding is an extremely selective process; it depends on the shape, size, constitutional makeup, and physicochemical properties of both drug and its target.^33^ We have calculated total 127 features and all the features are very common, so they can be effortlessly calculated for any new or unexplored protein or ligand listed in ESI S2.†

### For proteins

Global features refer to the features considering the entire protein. We have used the entire protein instead of features of only pockets and cavities. Our aim is to train a machine with the features that are easily calculable using merely the receptor. Calculating features of the cavity demands the information of the cavity. For the cavity information, we either need to have a co-crystallized structure of a protein with its ligand or we can try *in silico* methods. Both of the methods are well known but are time-consuming, here we are presenting a method in which can skip a few steps and reach the binding energy prediction relatively faster.

#### Amino acid sequence

The unique amino acid sequence of one protein is often referred to as its primary structure. Protein primary sequence is guided and specified by nucleotides present in the gene. Each amino acid is encoded by particular triplet set of codons. Native conformation of a protein is determined by the interatomic interactions along with the amino acid sequence, in a given environment.^34^ Chemical reactivity of an individual protein is defined by the type and spatial orientation of surface accessible amino acid side chains.^35^ Conformation, therefore, determines protein function, especially its interaction with ligand. Consequently, knowledge of primary sequence might play a crucial role to predict conformation as well as its interactive properties.

#### Protein secondary structure

Proteins secondary structure, stabilised through the local interactions among the adjacent residues are giving rise to a particular geometry by repetitive approach. Instead of considering coordinates of each atom present in the system, in order to minimise the computation time, we have selected a few special characteristics functional features (*e.g.* molecular weight, number of chains, number of ss bridges, and number of various types of secondary structure like helices, sheets; as mentioned in Table 1 of the protein of interest to build a prediction model towards feasibility of protein–ligand interaction. These features were calculated using programme DSSP.^36^

**Table 1:** Features details and their source

Molecule	Features	Source
Protein	Amino acid percentage	Calculated from Fasta files^31^
	Accessible surface of protein	DSSP^36^
	Number of hydrogen bonds in antiparallel bridges and parallel bridges	
	Number of hydrogen bonds of type O(I) → H–N(I-5), O(I) → H–N(I-4), O(I) → H–N(I-3), O(I) → H–N(I-2), O(I) → H–N(I-1), O(I) → H–N(I+0), O(I) → H–N(I+1), O(I) → H–N(I+2), O(I) → H–N(I+3), O(I) → H–N(I+4), O(I) → H–N(I+5)	
	Number of chains	
	Number of ss bridge	
	Number of residues	
Ligand	Atom count: C, N, O, H, S, P, Cl, F, Br, I	Padel descriptors^42^
	Bond count: number of single, double, triple bond including and excluding hydrogens	
	Ring count: number of 3, 4, 5, 6, 7, 8, 9 atom/carbon rings, aromatic rings, fused hetero rings, fused homo ring	
	Physicochemical properties: complexity, log *p*, hbond donor, hbond acceptor, topological surface area, mol. wt	Pubchem^41^

#### Accessible surface area

Solvent plays a crucial role in the interactions of proteins with their ligands. Solvent-accessible surface area (SASA) is the area of the protein that is directly in contact with solvent.^37^ Interaction of protein with ligand generally involves an entropically favored displacement of solvent molecules from the protein and ligand surfaces and an enthalpically favored reorganization between the protein and ligand along with the solvent molecule.^38^ SASA of the receptor is also calculated from DSSP programme.^36^

### For ligands

A drug sweeps thorough blood vessel, gastrointestinal fluids, small intestine before reaching its active site. As Lipinski *et al.*^39,40^ explained, a drug molecule must have the absorption, distribution, metabolism, excretion, and toxicity (ADMET) properties so as to qualify as a successful candidate. All these responses of a chemical compound are intrinsic and is a result of the combination of its various physical and chemical properties. Therefore, for defining a drug we have included all its physicochemical properties available in pubchem^41^ along with that few structural properties, which were calculated using a tool Padel Descriptor.^42^ We have included major 2-d properties of a small molecule along with physicochemical properties which define a molecule and differentiate it with others. List of features and their source is represented in Table 1 and details of the features are also listed in ESI S2.†

We haven't included intermolecular interaction features as we are going to use this prediction method in the very initial stage, prior to the formation of protein–ligand complex and considering the fact that binding intermolecular distance or constitution can only be generated using the complex structure. The presence of the complex structure validates that either computational molecular docking or experiment is already done, hence no need to get the binding affinity using this prediction model.

### Prediction models

Weka v3.8.0 (ref. 25 and 26) was used in our study. 2864 instances with 128 features were trained using Gaussian process,^43^ linear regression,^44^ multilayer perceptron,^45,46^ SMO regression,^47,48^ K-star,^49^ and Random forest.^50^ It is tested for 10-fold cross-validation. *i.e.* for each fold there are 286 instances for testing while rest 2578 are used for training. For the next fold another 286 instances are selected for testing and the rest used for training.

### Random forest model

Random forest (RF), introduced by Breiman^50^ is based on bagging *i.e.* bootstrap aggregation. It divides the entire dataset into subsets and builds a random tree for each subset which is called bootstrap sampling and runs prediction test on each sample tree, the final prediction result is the amalgamation of prediction of each Random Tree. In addition to bagging, RF splits the dataset on features. Each tree will be trained on a minimum of features that is *K*. The entire training dataset is *N* in number and there will be *I* number of random trees. For constructing a tree, a node is selected at random from the features set and growing the tree, each parent node is split into daughter nodes on the basis of best split considering information gain that is needed to be present in the data of the node.Information gain = entropy (parent node) − [average entropy (daughter node)]where entropy = −∑*p*_*i*_ log_2_ *p*_*i*_

Moreover, *p* is the probability of class.

### Gaussian process model

In a multivariate data, any point in space is a vector *x⃑* having components *X*_1_, *X*_2_, …*X*_*n*_. Gaussian Process (GP)^43^ by definition is a collection of random variables of any finite number which have consistent joint Gaussian distribution which is fully specified by its mean function (*μ*) and covariance function (ref).

This can be represented as
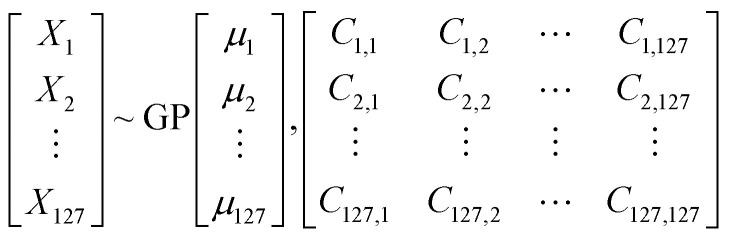
where *X*_1_, *X*_2_…, *X*_*n*_ are the components of vector *x⃑* which describe the features while *μ*_1_, *μ*_2_… *μ*_*n*_ are component of *μ⃗* which is the mean of corresponding feature. The matrix is the covariance matrix, each of its diagonal elements are variance of corresponding feature whereas the rest elements are their respective covariance. Covariance function characterizes correlations between different points in the space.

For making prediction utilizing Gaussian Process, we have used various kernels. Kernels basically calculate how 2 points 
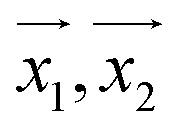
 in space are related which is termed as the covariance here. We have studied polykernel, normalised polykernel, and RBF kernel.

#### Polykernel

Polynomial kernel looks into the similarity of two input vectors on the basis of their dot product of the vectors. For a *p* degree of polynomial, it is defined as 
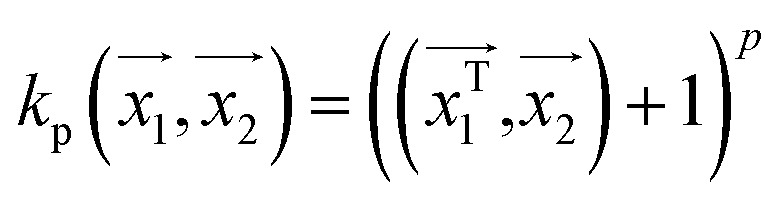
. In weka the parameter exponent controls the degree of polynomial. The default degree is set to 1, however we have toggled that to find a function which best fits our data.

#### Normalised polykernel

It is an extension of polykernel. This is defined as 
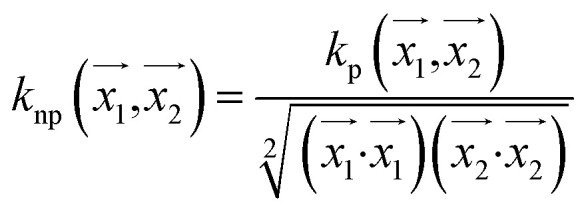
, the parameter exponent is same as the polykernel.

#### RBF kernel

Radial basis function (RBF) kernel uses the squared Euclidean distance function between two feature vectors in space. This can be defined as 
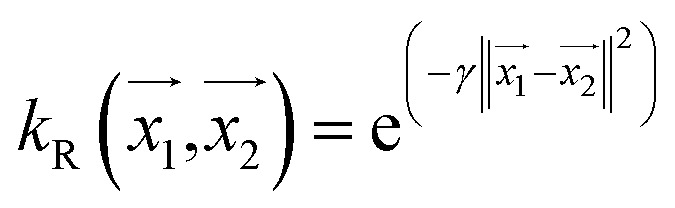
, where the parameter gamma is 0.01 by default.

### Multilayer perceptron model

Multilayer perceptron (MLP)^45,46^ model is an Artificial Neural Network model. Neural Network mimics biological neurons. Each element of input vector can be seen as single dendrite which have the information and it passes the information to the perceptron neuron. The perceptron forms a linear combination of inputs and their weights to computes an output and then the output calculation continues through an activation function. The classifier uses back propagation error to find the optimised weights. These perceptron makes up the hidden layer, there are more than one perceptron in a multilayer perceptron model to fit non-linearly separable data. Number of hidden layers can be defined in weka's MLP function.

### SMO model

SMO model in weka 3.6.8 (ref. 25 and 26) is based upon sequential minimal optimisation (SMO) algorithm^47^ for training a support vector classifier. Support vector machines (SVM) are learning algorithms that finds a hyperplane which separates the features of multiple classes of data. The points that are closest to the separator have nonzero weights and the rest have zero. The points with nonzero weights are called the support vectors because they hold up the separating plane. SVM uses kernel (same as Gaussian Process model), it implicitly maps the original data to a feature space of possibly infinite dimension in which data which is not separable in the original space becomes separable in the feature space. SMO algorithm quickly solves the SVM quadratic problem without any extra matrix storage and without invoking an iterative numerical routine for each sub-problem.

### Model evaluation

Internal 10-fold cross-validation and a blind set validation was implemented for prediction of binding affinity of protein–ligand pair. Cross-validation allows each instance of the dataset to be tested once for prediction, hence it is purely an unbiased basis for testing efficiency of a model. The performance of each model was evaluated using correlation coefficient (*R*^2^) and Root Mean Square Error (RMSE).Error (*E*) = actual binding affinity (*A*_A_) − predicted binding affinity (*A*_P_)

Mean actual binding affinity,
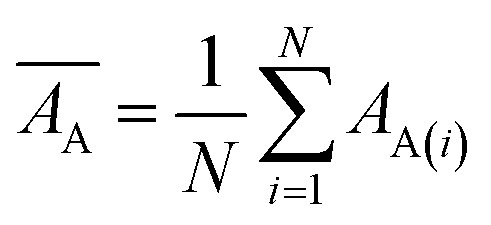


Mean predicted binding affinity,
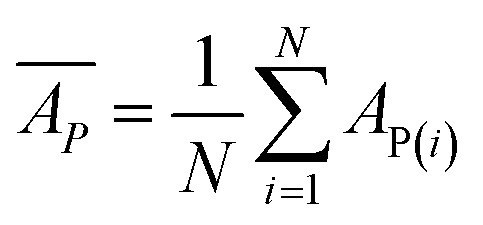

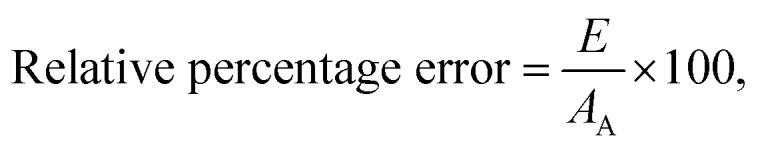


Mean error,
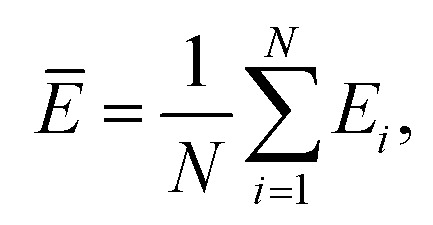

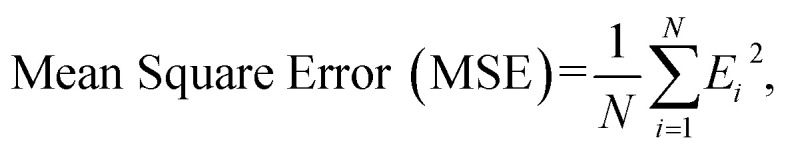

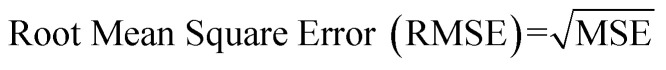

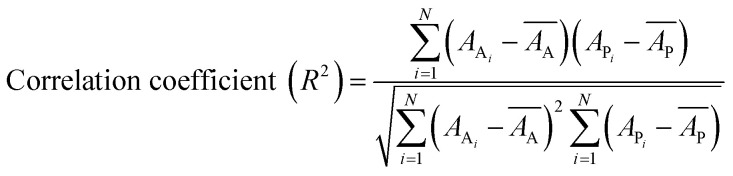


### Comparative study

In addition to testing the prediction models on our own dataset, we have drawn a comparison using our features with few of already published dataset. Xue *et al.*^24^ used 94 drugs against human serum albumin and have used SVM models for prediction the binding affinity. Deng *et al.*^27^ has used 105 diverse protein–ligand complexes and Wang *et al.*^29^ used pdbbind version 2012. Wang *et al.*^29^ had compared their model with various state of art prediction models with their Random forest model and concluded their model outperformed others. We have used the same list of proteins and ligands used by these authors and extracted our list of features for them. They have also tested their model on the basis *R*^2^ and RMSE. We have chosen their best *R*^2^ value for comparing with our result.

## Results

### Performance analysis

Machine learning algorithm incorporated in Weka 3.6.8 package^25,26^ has been used. It has some default parameters; however, we have also tried to optimise parameters according to the need of our dataset, and hence we are able to predict our binding energy with better correlation and lesser error. Below we present a summary of the regression algorithms used.

### Random forest

Random forest is an ensemble of various Decision Trees.^50^ The number of trees to be generated can be defined by the user. Weka's default value of the number of tree generation is 100. We have changed the parameter and recorded the correlation coefficient for each model. We have observed an increase in the correlation coefficient with the increase in number of trees; however, it is not increasing much after 300, so we have used 400 trees for our subsequent determination of number of features to be used per tree.

**Fig. 1:**
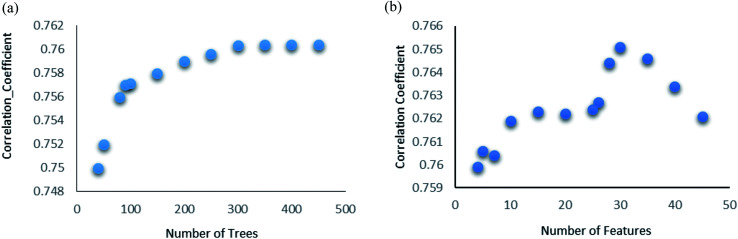
(a) Random forest algorithm's performance analysis. Change in correlation coefficient with change in number of trees. (b) Random forest algorithm's performance analysis. Change in correlation coefficient with change in number of features with number of iterations fixed at 400.

**Fig. 2:**
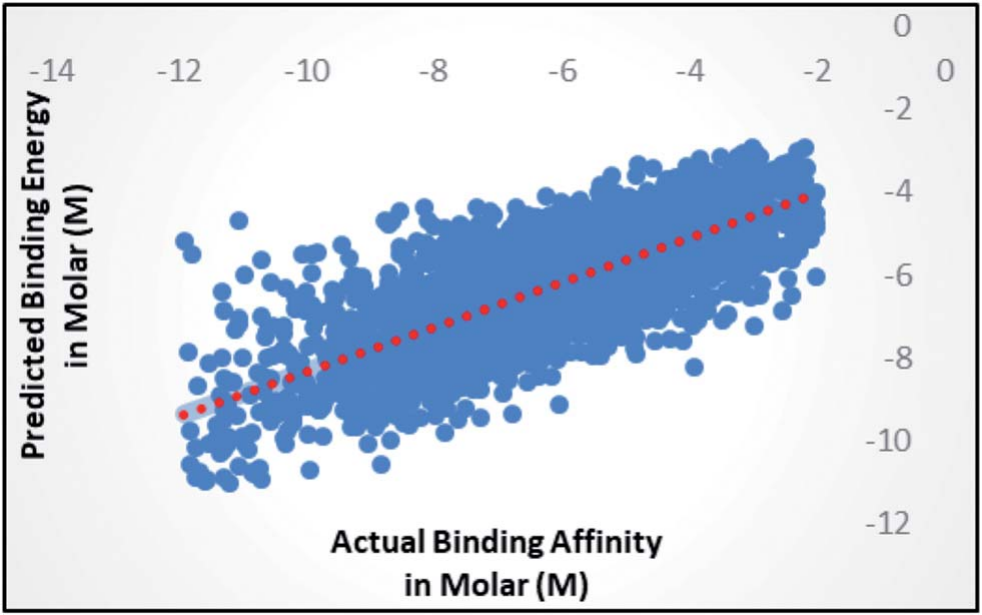
Scatter plot for actual *vs.* predicted binding affinity of v2015 dataset using Random forest with 400 iterations having 30 features in each.

When the number of features is set to 0 (default setting), then the actual number of features is calculated as log_2_((127) + 1) = 7. We observed that 400 iterations with 30 features in each iteration gave the best result, shown in Fig. 1a and b, with *R*^2^ = 0.7651 and error 1.31 molar (M). Variance was fixed at default 0.001 and the number of instances per leaf was set to 1 to reduce the probabilistic conditions. The correlation plot of actual *vs.* predicted binding affinity using the above-mentioned parameters is shown in Fig. 2.

### Gaussian process

**Fig. 3:**
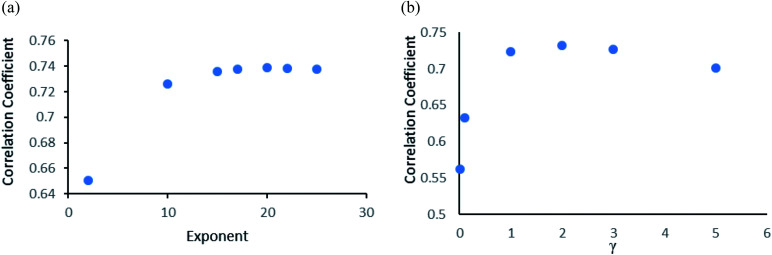
(a) Gaussian process algorithm's performance analysis. Change in the correlation coefficient with change in exponent value of normalised polykernel, (b) change in the correlation Coefficient with change in *γ* value of RBF kernel.

Gaussian process^43^ regression models utilizing normalised polykernel and RBF (Radial Basis Function) kernels were used in our study. In normalised polykernel, the degree of polynomial is assigned by a parameter named exponent. Weka 3.6.8 (ref. 25 and 26) package have 2.0 as default exponent, using that we got correlation coefficient 0.6505 and RMSE 1.53, we kept increasing stepwise and finally observed 20-degree polynomial gave the best results, correlation coefficient 0.7386 and RMSE 1.36 (Fig. 3a). In RBF kernel the parameter *γ* was set to 0.01 which gave correlation coefficient 0.5626 and RMSE 1.57. We further adjusted the parameter to get better results. When *γ* is set to 2, it gave the best results with correlation coefficient 0.7327 and RMSE 1.38 (Fig. 3b).

### Other models

**Fig. 4:**
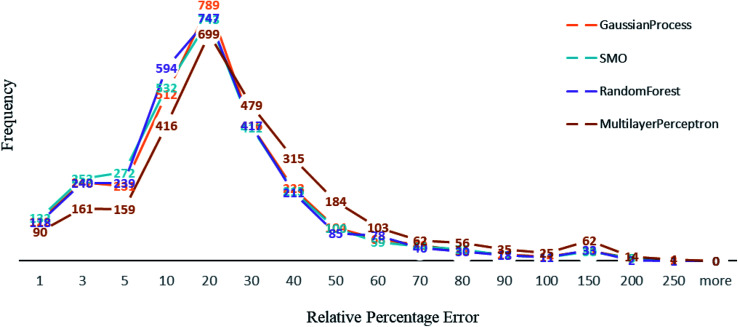
A comparison of percentage of relative error among algorithms used.

Other than Random forest and Gaussian Process, we also used multilayer perceptron, SMO, and K-star prediction models. multilayer perceptron and Linear regression did not suit our data well. We observed that the maximum number of instances have relative error less than 20%. Out of 2864, the number of instances that gave relative error less than 20% is 1938, 1932, 1896 and 1525 respectively in Random forest,^50^ SMO,^47^ Gaussian process^43^ and multilayer perceptron^45,46^ (Fig. 4). For less than 250 instances, we are getting more than 50% relative error. SMO and Random forest models are equally good with our data, however a slight difference is that 2 instances had 200% relative error in Random forest model and the number is 8 for SMO and 9 for Gaussian Process. This makes Random forest model better than the rest. We can see that multilayer perceptron is not performing well, 361 instances had relative error more than 50%. Out of 2864 instances, 2570 instances had error less than 0.2 log units of actual energy, which implies a significant prediction result.

### Comparative results

We have fine-tuned the algorithms to find out the best performance. We have observed that Random forest^50^ is outperforming all other algorithms. Wang *et al.*,^29^ Deng *et al.*^27^ and Xue *et al.*^24^ studied prediction of protein–ligand binding affinity. We have used the instances from their datasets and calculated the features that we used in our study.

**Fig. 5:**
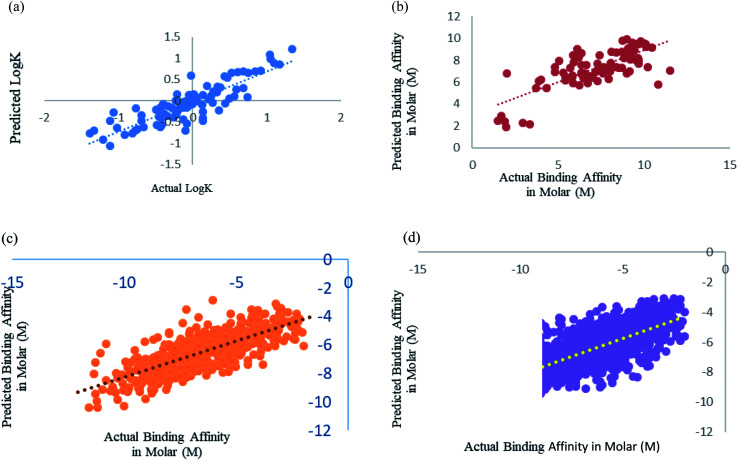
(a) Scatter plot for actual *vs.* predicted log *K* of Xue's dataset using SMO utilising RBF kernel over 10-fold cross-validation. (b) Scatter plot for actual *vs.* predicted binding affinity of Deng's dataset using Random forest over 10-fold cross validation. (c) Scatter plot for actual *vs.* predicted binding affinity of Wang's dataset using Random forest. (d) Scatter plot for actual *vs.* predicted binding affinity of Kramer's dataset using Random forest.

Xue *et al.*^24^ had used 94 drugs against human serum albumin, we have also used human serum albumin against 91 drugs as 3 drugs were obsolete at present and we could not find their information. Xue *et al.*^24^ used Support Vector Machine (SVM) regression using RBF kernel for their study, so for this dataset we have also used SMO models, which is the SVM regression algorithm incorporated in Weka 3.6.8.^25,26^ We got a better correlation coefficient 0.86 as compared to their reported correlation coefficient 0.63 for 10-fold cross-validation (Fig. 5a). They have trained the machine for drugs against single protein human serum albumin, we can conclude that ML models can predict protein–ligand interaction with one protein and many ligand with very minimal error, RMSE 0.114.

Deng *et al.*^27^ were one of the initiators of carrying out prediction study over diverse class of proteins. They had used 105 diverse protein–ligand complex and we also have used the same complexes with our calculated features. We have used Random forest prediction models^50^ for prediction test. We got a better correlation coefficient 0.75 as compared to their reported correlation coefficient 0.64 for 10-fold cross-validation (Fig. 5b).

Wang *et al.*^29^ used the PdbBind Database^30^ of the year 2012 for their study and had reported an appreciable prediction results. We have used the exact same protein ligand pairs in test and training set as used by them. We have used Random forest Model^50^ for the dataset, as Wang *et al.*^29^ concluded that the Random forest models outperformed others. We got a better correlation coefficient 0.75 and RMSE 1.29 as compared to their reported correlation coefficient 0.67 and RMSE 1.46 (Fig. 5c). Other than the generic dataset used by Wang we have also compared family specific dataset used by them.

Kramer and Gadeck^28^ had also used the PdbBind Database^30^ of the year 2009. They have used refined set of protein–ligand binding data which comprised 1741 complexes out of which they had excluded the complex in which the ligands which were polymer, peptides and ATPs. We have also eliminated those and 1387 complexes were used. Random forest algorithm with 400 iterations and 30 features in each iteration was used to predict. Prediction was analyzed using 10-fold cross validation (Fig. 5d). As they had already drawn a comparison among other models and programs which are used in prediction and stated their performance was significantly better, so we have considered their method as benchmark for our comparison an observed our method and features outperformed theirs.

**Fig. 6:**
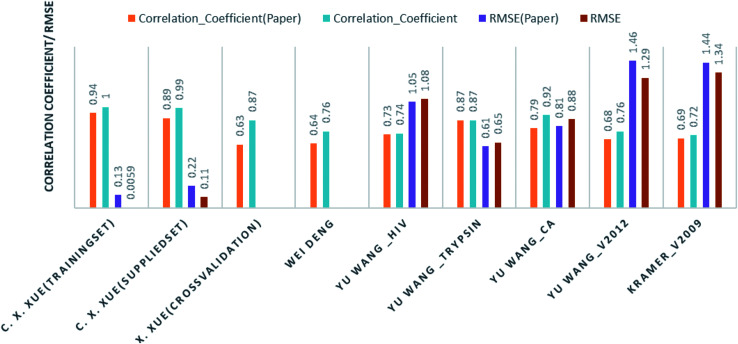
Bar graph for the correlation coefficient and RMSE (both rounded off to two decimal places) of the prediction models. A comparison of results using our prediction model and the results published by the respective authors.

Comparison of our results with the published one is listed in Table 2 and represented in Fig. 6.

**Table 2:** Comparison table

Authors	Training instances	Test/method	Their result	Our result
C. X. Xue, *J. Chem. Inf. Comput. Sci.*, 2004 (ref. 24)	Human serum albumin; 95 drugs	Training set	*R* ^2^ = 0.94: RMSE = 0.134	***R*** ^**2**^ = **0.1; RMSE** = **0.0059**
		Supplied test set	*R* ^2^ = 0.89: RMSE = 0.222	***R*** ^**2**^ = **0.987: RMSE** = **0.114**
		Cross validation	*R* ^2^ = 0.63	***R*** ^**2**^ = **0.867**
Wei Deng, *J. Chem. Inf. Comput. Sci.*, 2004 (ref. 27)	105 (diverse) complexes	Cross validation	*R* ^2^ = 0.64	***R*** ^**2**^ = **0.756**
Christian Kramer and Peter Gedeck, *J. Chem. Inf. Model.*, 2011 (ref. 28)	Pdbbind v2009; 1387 complexes	Cross validation	*R* ^2^ = 0.69	***R*** ^**2**^ = **0.72**
Yu Wang, *J. Comput.–Aided Mol. Des.*, 2014 (ref. 29)	Hiv protease 136	Supplied test set 34	*R* ^2^ = 0.728: RMSE = 1.05	RF: *R*^2^ = 0.69: RMSE = 1.07
				**DT: *R*** ^**2**^ = **0.74: RMSE** = **1.08**
	Trypsin 88	Supplied test set 22	*R* ^2^ = 0.871: RMSE = 0.61	RF: *R*^2^ = 0.85: RMSE = 0.69
				**Kstar: *R*** ^**2**^ = **0.873: RMSE** = **0.65**
	Carbonic anhydrase 100	Supplied test set 26	*R* ^2^ = 0.790: RMSE = 0.92	***R*** ^**2**^ = **0.8078: RMSE** = **0.8867**
	V2012 2318	Supplied test set 579	*R* ^2^ = 0.678: RMSE = 1.46	***R*** ^**2**^ = **0.7564: RMSE** = **1.299**

### Blind set validation

**Fig. 7:**
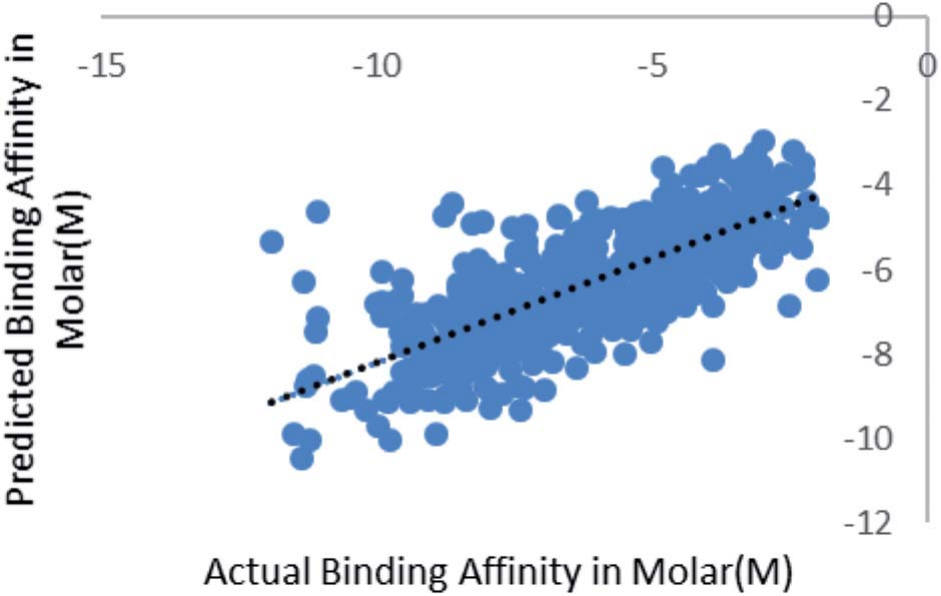
Scatter plot for actual *vs.* predicted binding affinity of external dataset using Random forest.

We have also validated our prediction model using external test set. Out of the 2864 instances we have randomly sampled 80% of the data in training set and rest 20% in the test set. Model was trained with 2291 instances and was supplied with 573 test instances. The blind validation also gave promising results, *R*^2^ = 0.75 and RMSE = 1.38. The result is represented in Fig. 7.

### Negative set validation

The algorithm and features were also validated against non-binders. Non-binding or decoy data was used from the DUD-E database.^51^ We have used 12 proteins with pdb id (1e66, 2oi0, 3d0e, 1bcd, 3odu, 3ccw, 3g0e, 2ojg, 3bgs, 2azr, 1ype, 1sqt) from the DUD-E database which contribute in 2249 non-binders and 1110 binders. The same features were calculated for all the binders and non-binders. Random forest algorithm was trained using 10-fold cross validation in prediction. The prediction results are shown in Table 3. The correctly classified instances are 3250 means 96.755% accuracy and incorrectly classified instances 109 3.245%.

**Table 3:** Result of prediction of feasibility of protein–ligand interaction

TP rate	0.968
FP rate	0.056
Precision	0.968
Recall	0.968
*F*-Measure	0.967
MCC	0.927
ROC area	0.994
PRC area	0.994

### Feature selection

We have used 127 features of protein and ligand for training. We have further explored and employed attribute selection filter of Weka package^25,26^ which ranks the attributes on the basis of their information gain. Out of 127, we have observed a set of 93 attributes giving the best prediction results shown in Table 4. However, there is not significant difference in the results, so top 18 (9 of ligand and 9 of protein) attributes can be used. Ranking list of the attributes is given in ESI 4.†

**Table 4:** Change in true positive rate of protein binding prediction using Random forest algorithm with respect to decrease in number of features

Number of attributes	TP rate
127	0.968
93	0.969
82	0.965
72	0.966
64	0.967
58	0.966
54	0.968
48	0.965
38	0.963
28	0.96
18	0.96
10	0.93

## Conclusion

Discovery of potential drug or lead through protein–ligand interaction is a herculean task. As the interaction between specific ligand and protein depend on some characteristic features, determining a particular feature of ligand and protein plays a crucial role in identifying the interaction. The aim of our study is to predict whether an unknown ligand can interact with a protein, which may be utilised as a potential lead. Towards this end we exploit the binding energy in terms of dissociation constant *K*_d_ and inhibition constant *K*_i_ using Machine Learning algorithms with a few significant features of interaction. This method reduces the running time in comparison of state-of-the art computational techniques. Out of various machine learning algorithms like multilayer perceptron, SVM and Gaussian Process, Random forest model best suited the protein–ligand binding energy prediction problem. RF model's performance is highly dependent on the number of iterations (trees) and number of features used to build each tree, on the other hand machine learning relies upon the number and significance of features on which model is trained upon. Use of too many non-significant features may cause the machine to learn fuzzy patterns, leading to poor prediction. We have performed extensive experimentation in order to select optimum parameters of the ML algorithms which not only reduced the run time but also increase the accuracy of prediction. Further comparative study revealed that our strategy and the RF-model perform much better in the diverse dataset towards prediction of the unknown interaction. These models have the potential to identify the binding site for the interaction of protein and ligand based on their structural, physicochemical, and coordinate features.

## Conflicts of interest

There are no conflicts to declare.

## Supplementary Material
